# Supplementary material for: CCP1, a Regulator of Tubulin Post-Translational Modifications, Potentially Plays an Essential Role in Cerebellar Development
Source: Int J Mol Sci. 2023 Mar 10;24(6):5335. doi: 10.3390/ijms24065335 (PMC10049023; doi:10.3390/ijms24065335)
Supplement: Supplementary file 1 [file ijms-24-05335-s001.zip › ijms-2250445-supplementary.pdf]

Figure S1.

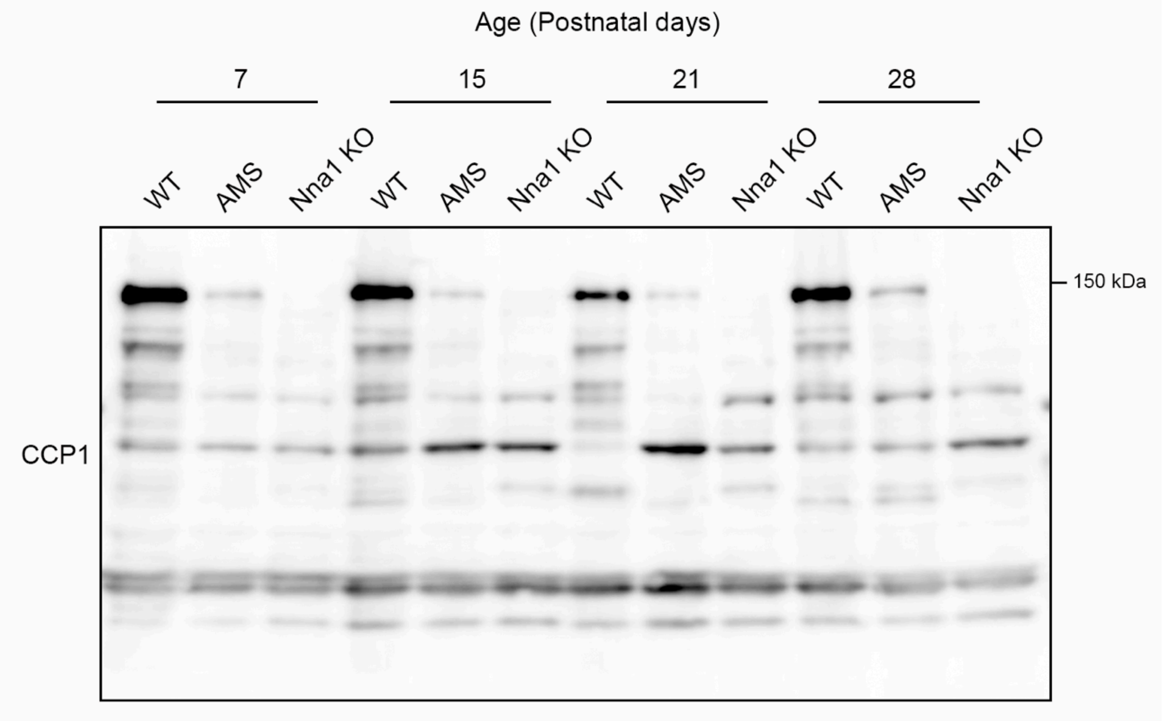

Figure S1. The CCP1 protein expression in the cerebellar cortex in four age groups of WT, AMS and Nna1 KO mice. Many protein fragments of different sizes are observed except for the purpose protein. CCP1: cytosolic carboxypeptidase. AMS: ataxia and male sterility.

Figure S2.

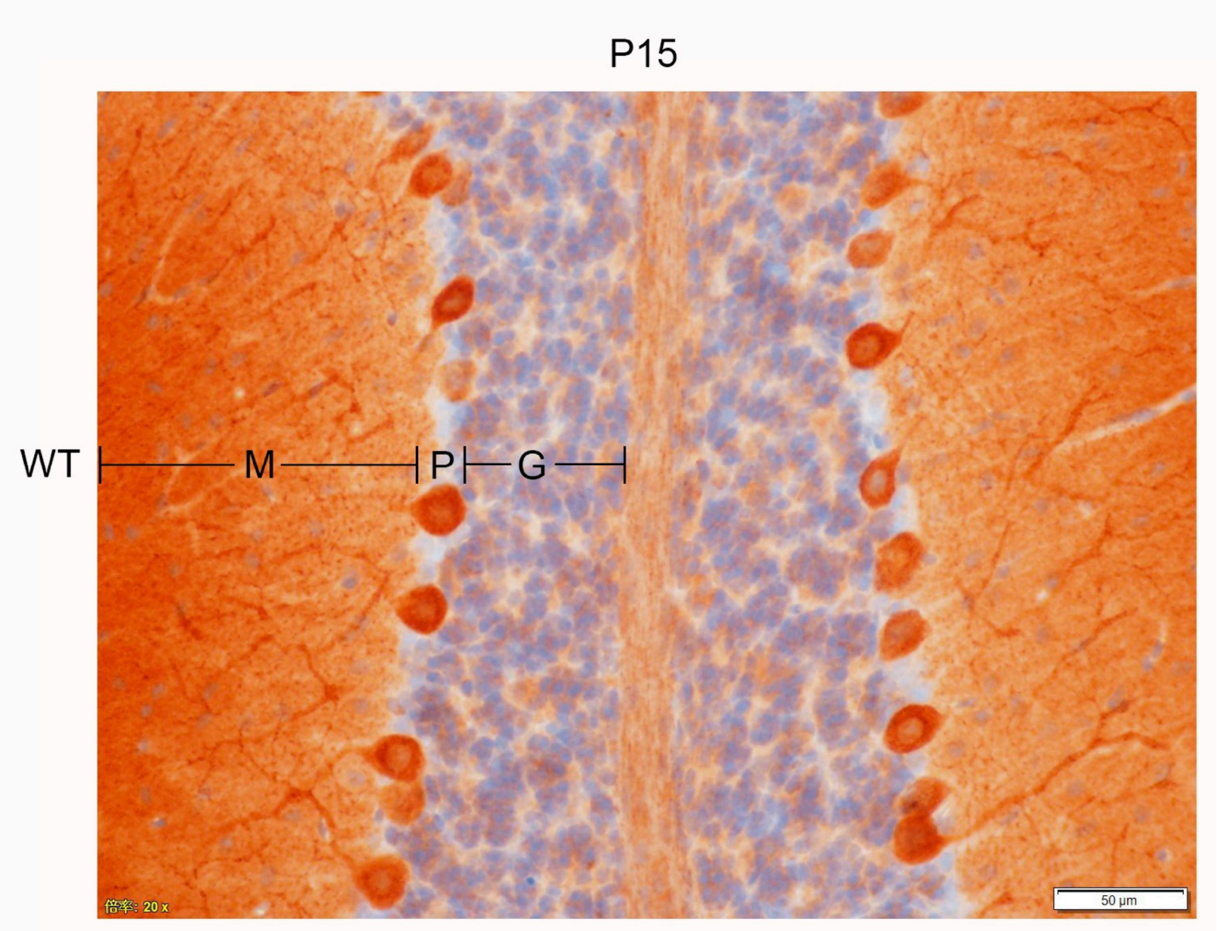

Figure S2. The representative light microscope appearance of the cerebellum at postnatal day (P) 15 of WT mouse. CCP1 is expressed in the soma and dendrites of Purkinje cells. M, molecular layer; P, Purkinje cell layer; G, granular layer; Scale bars: 50 μm. CCP1: cytosolic carboxypeptidase.
